# Defining the gene repertoire and spatiotemporal expression profiles of adhesion G protein-coupled receptors in zebrafish

**DOI:** 10.1186/s12864-015-1296-8

**Published:** 2015-02-08

**Authors:** Breanne L Harty, Arunkumar Krishnan, Nicholas E Sanchez, Helgi B Schiöth, Kelly R Monk

**Affiliations:** Department of Developmental Biology, Washington University School of Medicine, St. Louis, MO 63110 USA; Department of Neuroscience, Functional Pharmacology, Uppsala University, BMC, Box 593, 751 24 Uppsala, Sweden; Hope Center for Neurological Disorders, Washington University School of Medicine, St. Louis, MO 63110 USA

**Keywords:** Adhesion G protein-coupled receptors, Zebrafish genome, Expression profiling, High-throughput quantitative real-time PCR

## Abstract

**Background:**

Adhesion G protein-coupled receptors (aGPCRs) are the second largest of the five GPCR families and are essential for a wide variety of physiological processes. Zebrafish have proven to be a very effective model for studying the biological functions of aGPCRs in both developmental and adult contexts. However, aGPCR repertoires have not been defined in any fish species, nor are aGPCR expression profiles in adult tissues known. Additionally, the expression profiles of the aGPCR family have never been extensively characterized over a developmental time-course in any species.

**Results:**

Here, we report that there are at least 59 aGPCRs in zebrafish that represent homologs of 24 of the 33 aGPCRs found in humans; compared to humans, zebrafish lack clear homologs of *GPR110*, *GPR111*, *GPR114*, *GPR115*, *GPR116*, *EMR1*, *EMR2*, *EMR3*, and *EMR4*. We find that several aGPCRs in zebrafish have multiple paralogs, in line with the teleost-specific genome duplication. Phylogenetic analysis suggests that most zebrafish aGPCRs cluster closely with their mammalian homologs, with the exception of three zebrafish-specific expansion events in Groups II, VI, and VIII. Using quantitative real-time PCR, we have defined the expression profiles of 59 zebrafish aGPCRs at 12 developmental time points and 10 adult tissues representing every major organ system. Importantly, expression profiles of zebrafish aGPCRs in adult tissues are similar to those previously reported in mouse, rat, and human, underscoring the evolutionary conservation of this family, and therefore the utility of the zebrafish for studying aGPCR biology.

**Conclusions:**

Our results support the notion that zebrafish are a potentially useful model to study the biology of aGPCRs from a functional perspective. The zebrafish aGPCR repertoire, classification, and nomenclature, together with their expression profiles during development and in adult tissues, provides a crucial foundation for elucidating aGPCR functions and pursuing aGPCRs as therapeutic targets.

**Electronic supplementary material:**

The online version of this article (doi:10.1186/s12864-015-1296-8) contains supplementary material, which is available to authorized users.

## Background

The G protein-coupled receptor (GPCR) superfamily comprises the largest class of cell membrane receptors found in metazoan proteomes [1]. In humans, more than 800 genes encoding different GPCRs have been identified and phylogenetically divided into five discrete families: **g**lutamate, **r**hodopsin, **a**dhesion, **f**rizzled/taste2, and **s**ecretin (*GRAFS* classification) [2]. Adhesion GPCRs (aGPCRs) are the second largest of the five GPCR families, with 33 and 31 members in humans and mice, respectively [3]. The aGPCRs are further subdivided into nine groups based on phylogenetic analysis of the 7-transmembrane domain (7TM) [4]. Although members of this family follow the same general structural pattern as other GPCRs, they differ in that they are characterized by an extremely long N-terminus that contains the GPCR autoproteolysis-inducing (GAIN) domain, [5] which encompasses the highly conserved GPCR proteolytic site (GPS). Most aGPCRs undergo autoproteolysis at the GPS motif, which results in a protein that is separated into an N-terminal fragment (NTF) and C-terminal fragment (CTF) that are thought to remain non-covalently attached at the cell surface [6]. The “adhesion” classification was given to this family of GPCRs due to the large number of classical cell adhesion domains found in the NTFs of many of these receptors [4,7]. In other proteins, these “adhesion” domains (*e.g.*, EGF-like domains and cadherin domains) are involved in protein-protein, cell-matrix, and cell-cell interactions, leading to the idea that they perform similar functions in aGPCRs [7]. Recent data for multiple aGPCRs suggests that these proteins can function as adhesion molecules by virtue of the NTF, and as classical GPCRs that signal through G-proteins by virtue of the CTF, in addition to the roles the NTF and CTF have in concert with one another [8-13].

In addition to their protein domain complexity, aGPCRs have been difficult to study due to their large size and complex genomic structures, with many small exons separated by very large introns [4]. Additionally, aGPCRs have numerous splice isoforms, often lacking one or more protein domains in the NTF, which may have functional or regulatory roles [14]. The study of aGPCRs is further complicated by the fact that this family is identified primarily based on structural similarity at the protein level because, on a sequence level, aGPCRs can be extremely divergent from one another [4]. However, despite the divergence between family members, aGPCRs in general are evolutionarily ancient and highly conserved, with a homolog found in social amoeba, *Dictyostelium discoideum* [15].

In recent years, the zebrafish (*Danio rerio*) has become a premiere model organism for the study of a wide variety of physiological processes and disease states during development and in adult animals [16,17]. Moreover, zebrafish have proven to be useful models to study aGPCRs, especially in the context of development. For example, the functions of Gpr126 in Schwann cell myelination and inner ear morphogenesis were discovered in zebrafish [18,19]. Zebrafish studies also demonstrated that multiple aGPCRs - Celsr1a [20], Celsr1b [20], Celsr2 [21], and Gpr125 [22] - are critical modulators of planar cell polarity during vertebrate gastrulation. Additionally, Celsr3 is essential for normal development of visual circuitry in the zebrafish retina [23]. Although advances have been made in understanding aGPCR biology using zebrafish, the utility of this model has been impaired by the lack of a complete aGPCR repertoire and gene expression profiles. To address this deficit, we mined the *Danio rerio* genome to determine which aGPCRs are encoded in the zebrafish genome. We also performed qPCR to characterize aGPCR expression over a developmental time-course, as well as in a wide collection of adult tissues. Our studies demonstrate that there are at least 59 aGPCRs in zebrafish representing 24 of the 33 human aGPCRs, with similar expression profiles as their mammalian homologs.

## Results and discussion

### Defining the zebrafish aGPCR repertoire

To define the zebrafish aGPCR repertoire, we first compiled a list of nucleotide and protein sequences for 33 aGPCRs annotated in the Zv8 release of the zebrafish genome using three genomic databases: GenBank [24], Ensembl (release 75) [25], and the zebrafish model organism database (ZFIN) [26]. Next, we used genome alignment and search tools - BLAST [27], UCSC Genome Browser [28], and Sequencher (http://www.genecodes.com) - to further mine the zebrafish genome for additional predicted aGPCR sequences. We conducted BLAST [27] searches using both the nucleotide and amino acid sequences of all of the 33 previously annotated zebrafish aGPCRs, as well as aGPCR sequences from five additional species: stickleback (*Gasterosteus aculeatus*), mouse (*Mus musculus*), rat (*Rattus norvegicus*), dog (*Canis lupus familiaris*), and human (*Homo sapiens*). With this first pass of analysis, we obtained 40 putative aGPCRs encoded in the zebrafish genome.

Next, we took these 40 putative zebrafish aGPCR sequences and BLASTed them a second time against the zebrafish genome (Zv8). This step was essential for two reasons: 1) to determine if multiple predicted aGPCR sequences could be consolidated because they actually represented the same gene, and 2) to search for more divergent paralogous sequences (*e.g.*, gene duplicates). Indeed, further analysis of BLAST results suggested that several of the initial predicted aGPCR sequences might belong to the same genes. For example, one predicted sequence encoded the N-terminal domains and another encoded the GAIN and 7TM domains. In these instances, RT-PCR using primers overlapping both sequences was used to determine if sequences were indeed part of the same transcripts (data not shown). Similarly, it was important to determine if any of the initial 40 putative aGPCRs had paralogs because a whole genome duplication event occurred in the ray-finned fish lineage approximately 300 million years ago, which coincided with the radiation of teleost species [29-31]. Moreover, several lineage-specific tandem duplication events have occurred in zebrafish following the whole genome duplication event [29]. Therefore, many zebrafish genes are present in multiple copies. These searches resulted in a total of 50 predicted aGPCRs.

**Figure 1:**
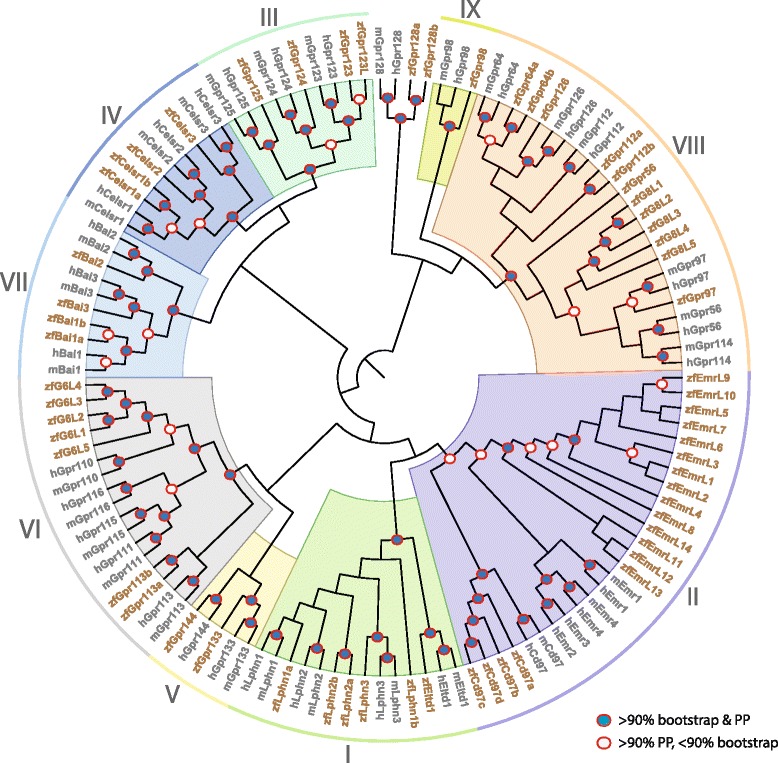
**Phylogenetic analysis of zebrafish, mouse, and human aGPCRs.** The evolutionary history of the zebrafish aGPCRs relative to their mammalian orthologs was inferred using the Maximum Likelihood (ML) method based on the JTT matrix-based model using MEGA5. The bootstrap consensus tree inferred from 1000 replicates is shown as a representation of the possible evolutionary history of the 7TM domain of zebrafish (zf), mouse (m) and human (h) aGPCRs. The topology inferred from ML analysis was also supported by Markov Chain Monte Carlo (MCMC) analysis, using the Bayesian approach implemented in MrBayes version 3.2. Blue circles with solid red outlines were shown for the nodes that had more than 90% confidence support from ML bootstrap analysis and Bayesian posterior probabilities (PP). White circles with solid red outlines denote nodes that had 90% PP support but less than 90% bootstrap support. Supports are only shown for nodes recovered by both ML and Bayesian inference, with BPP > 0.9 and bootstrap > 50%. Zebrafish gene identifiers/names are highlighted in brown text. It must be noted here that the topology supported by ML and Bayesian methods for Group 1 and Group VIII slightly differ from each other, although both methods recovered the overall clusters of Group 1 and Group VIII. The variations are that zfLphn1b and zfGpr56 are placed basal to their respective groups in the ML tree, however, with a relatively low bootstrap support. Nevertheless, the topology showing the homologous relationships of zfLphn1b and zfGpr56 with their mammalian counterparts are supported by Bayesian topology with PP > 90% (see Additional file 2).

Upon the release of Zv9, we performed additional searches using our previously defined list of 50 aGPCRs from Zv8, as well as the updated BioMart feature in Ensembl for all proteins with a GPS motif and an aGPCR-like 7TM domain. This second round of genome mining recovered an additional 9 aGPCRs, most of which appear to be zebrafish-specific. Our results indicate that there are at least 59 full-length aGPCRs in the zebrafish genome. Phylogenetic analysis suggests that they encode homologs for 24 of the 33 human aGPCRs, and that nine human family members, *EMR1*, *EMR2*, *EMR3*, *EMR4*, *GPR110*, *GPR111*, *GPR114*, *GPR115*, and *GPR116* do not appear to have clearly defined homologs in zebrafish (Figure 1). Consistent with the evolutionary history of zebrafish, we determined that 13 of the 24 zebrafish aGPCRs that have human homologs are present in multiple copies. See Additional file 1 for all accession numbers.

Here we present complete nomenclature for these zebrafish homologs based on phylogenetic analysis. The aGPCRs we uncovered are referred to with gene names lower-case and italicized. Any gene present in multiple copies is denoted as the gene name followed by a letter, such as “*lphn1a*” and “*lphn1b*”. For the predicted aGPCR sequences that appear to be members of zebrafish-specific expansions that are not clear homologs of any human aGPCRs, we named them based on the group that they cluster with phylogenetically followed by a number (*e.g.*, zfG8L1 is most phylogenetically similar to aGPCRs in Group VIII). However, we note that further analysis is necessary to be sure of the true identity of some of the predicted aGPCR sequences. The phylogenetic analysis of the 7TM regions shows how the zebrafish aGPCRs either cluster closely with their mouse and human orthologs or into distinct zebrafish-specific clades (Figure 1). Separate phylogenetic analyses were performed on each cluster in order to determine nomenclature of the genes that had unstable positioning in the overall analysis (data not shown). For example, zfLphn1b and zfGpr56 do not clearly cluster with their mammalian homologs in the overall analysis, but when Bayesian analysis was conducted on Groups I and VIII independently, these genes clearly cluster with their mammalian counterparts (Additional file 2). Importantly, the zebrafish aGPCRs cluster into nine groups in the same manner as was previously described for human and mouse aGPCRs [4,32].

**Figure 2:**
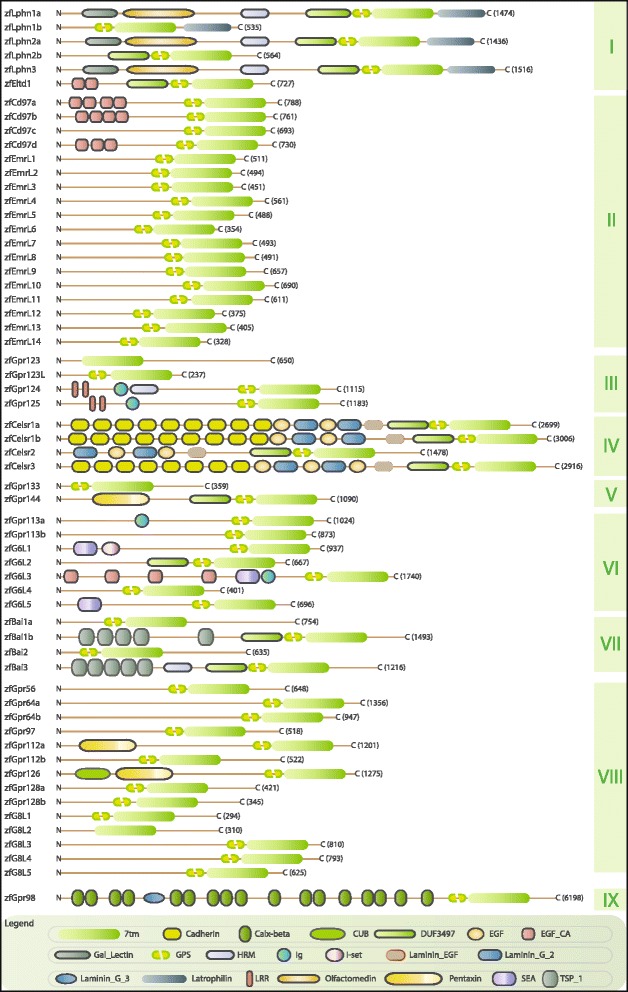
**Schematic drawing of domain organizations of aGPCRs encoded in the zebrafish genome.** aGPCR domain architectures were predicted using CD-search, Pfam, and InterProScan prediction algorithms. Each panel shows the long N-termini with multiple functional domains, seven integral transmembrane helices embedded in the membrane, and the intracellular C-terminal end for each zebrafish aGPCR. For display purposes, the length of the N-termini of each panel does not correspond to a measured scale of amino acids; instead, the overall length of the receptors is shown in parentheses at the C-terminal end of each cartoon. The depicted domains are: seven-pass transmembrane domain (7TM), Cadherin domain, Calx-beta domain (CALXβ), CUB domain (for complement C1r/C1s, Uegf, Bmp1), Domain of unknown function 3479 (DUF3479), Epidermal growth factor (EGF), Calcium-binding EGF domain (EGF_Ca), Gal_lectin, GPCR-proteolytic site (GPS), Hormone receptor motif (HRM), immunoglobulin domains (IG), Immunoglobulin I-set domain (I-set), Laminin EGF domain (Laminin_EGF), Laminin_G_2, Latrophilin C-terminal domain (Latrophilin), Leucine Rich Repeats (LRR), Olfactomedin, Pentraxin domain, SEA domain, and Thrombospondin type 1 domain (TSP_1).

The domain architectures of zebrafish aGPCRs (Figure 2) were predicted using the latest versions of Conserved Domain Search service (CD-Search) [33], Pfam search [34], and the InterProScan software package [35]. Although the phylogenetic tree shown in Figure 1 was built based on the protein sequence of the 7TM domains, most zebrafish aGPCRs also share relevant protein domains found in the N-termini of their mammalian counterparts (Figure 2). This includes the zebrafish-specific aGPCRs, as the predicted protein sequences share protein domains found in the N-termini of various members of their group, providing further support for the tree topology and nomenclature. For consistency, the domain architectures shown were made using the protein sequences in Ensembl, as these have been manually annotated. However, it is important to note that in some cases, the predicted protein sequences in GenBank are more complete, and may contain additional predicted domains that are not shown here, as they have not yet been confirmed. Additionally, as for many zebrafish genes, the coding domain sequences (CDS) of zebrafish aGPCRs are often incomplete. Therefore, it is difficult to determine if the differences in domain architectures of zebrafish versus mammalian aGPCRs are real differences or simply because the CDS are incomplete at the 5’ end and thus the domains have not yet been annotated.

Importantly, the only other GPCR subgroup with a defined repertoire in zebrafish is the trace amine GPCR group. Interestingly, trace amine GPCRs also underwent a large expansion relative to other vertebrates [36], similar to the aGPCR zebrafish-specific expansion we have described here. Similarly, our data are consistent with global characteristics of the zebrafish genome relative to the human genome. For example, 71.4% of all human genes have at least one ortholog in zebrafish, which is consistent with our finding that 72.7% of human aGPCRs have at least one zebrafish ortholog [37]. Further, of the human genes that have zebrafish orthologs, 47% of those are in a one-to-one relationship [37]. This is also consistent with our finding that 45.8% of the human aGPCRs are in a one-to-one relationship with their zebrafish ortholog.

### Expression profiling of aGPCRs in zebrafish

The expression profiles of aGPCRs have been previously determined in a collection of adult tissues in mouse [32], rat [32], and human [38]; however, to our knowledge, the expression of this family of GPCRs has never been extensively characterized throughout development, in any species. Additionally, expression profiles of the aGPCR repertoire in adult tissues are not resources currently available for the zebrafish research community. To address this, we defined the gene expression profiles of each of the zebrafish aGPCRs using a combination of high-throughput qPCR (for the first 50 genes identified in Zv8) [39] and conventional qPCR (for the remaining 9 genes identified later in Zv9). We chose 10 adult tissues to represent nearly every major organ system: brain, eye, heart, intestine, kidney, liver, skeletal muscle, skin, ovaries, and testes. We chose 12 developmental time-points to represent major milestones: 1 hour post-fertilization (hpf; cleavage period, ~4 cells), 3 hpf (blastula period; ~1000 cells), 5.3 hpf (early gastrulation), 10 hpf (late gastrulation, early segmentation), 14 hpf (segmentation), 24 hpf (most organ systems have formed), 3 days post-fertilization (dpf, larvae have hatched from chorions), 5 dpf (swimming), 7 dpf, 11 dpf, 14 dpf, and 21 dpf (juvenile stages defined by active hunting and rapid body growth). qPCR data was analyzed using the ΔΔCt method [40] (raw data is provided in Additional file 3). To control for starting input amount, we normalized all Ct values to a control gene, *importin-8* (*ipo8*), which showed stable expression in all time-points and tissues. We calculated fold change in expression relative to the 21 dpf time-point, as it represents a middle point between development and adulthood, where the fish has undergone all major developmental milestones and has acquired all tissues and organs, but is not yet a fully mature adult.

**Figure 3:**
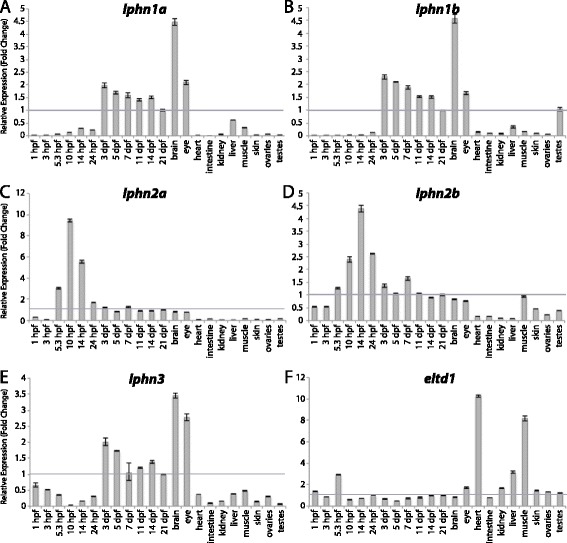
**Group I aGPCR expression data. (A-F)** Relative expression of zebrafish Group I aGPCRs obtained with high-throughput quantitative real-time PCR from a collection of 12 developmental time-points and 10 adult tissues. **(A)** *lphn1a*, **(B)** *lphn1b*, **(C)** *lphn2a*, **(D)** *lphn2b*, **(E)** *lphn3*, **(F)** *eltd1*. Error bars display the upper (RQmax) and lower (RQmin) limits of possible relative quantification values. Fold changes shown are relative to expression at 21 dpf (denoted by gray line at y = 1).

**Figure 4:**
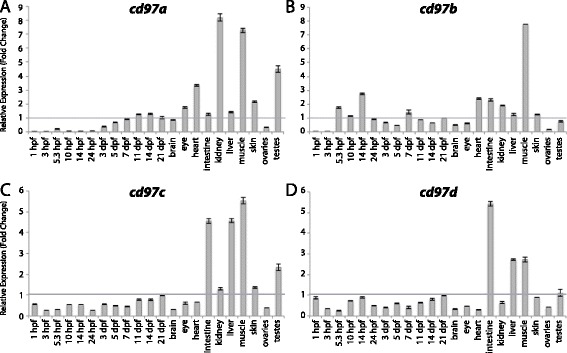
**Group II aGPCR expression data – part 1.** Relative expression of zebrafish Group II aGPCRs obtained with high-throughput quantitative real-time PCR in a collection of 12 developmental time-points and 10 adult tissues. **(A)** *cd97a*, **(B)** *cd97b*, **(C)** *cd97c*, **(D)** *cd97d*. Error bars display the upper (RQmax) and lower (RQmin) limits of possible relative quantification values. Fold changes shown are relative to expression at 21 dpf (denoted by gray line at y = 1).

**Figure 5:**
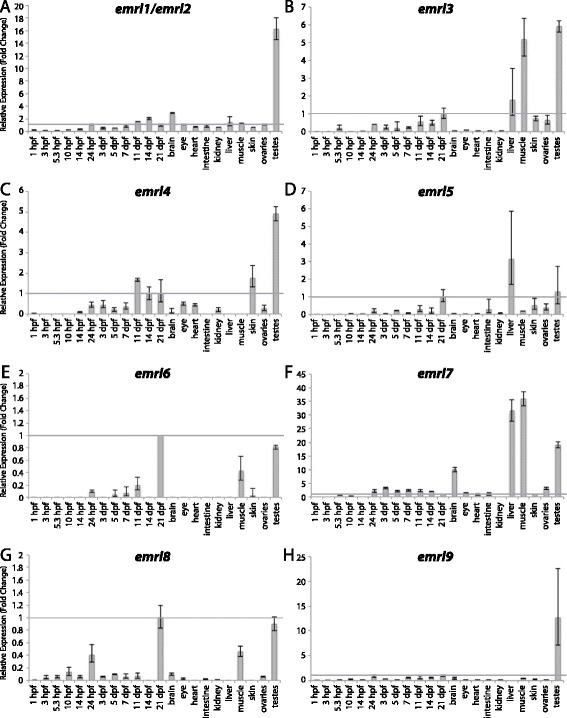
**Group II aGPCR expression data – part 2.** Relative expression of zebrafish Group II aGPCRs obtained with either high-throughput quantitative real-time PCR or conventional qPCR in a collection of 12 developmental time-points and 10 adult tissues. **(A)** *emrl1/emrl2*, **(B)** *emrl3*, **(C)** *emrl4*, **(D)** *emrl5*, **(E)** *emrl6*, **(F)** *emrl7*, **(G)** *emrl8*, and **(H)** *emrl9*. Error bars display the upper (RQmax) and lower (RQmin) limits of possible relative quantification values. Fold changes shown are relative to expression at 21 dpf (denoted by gray line at y = 1). *emrl1* and *emrl2* could not be distinguished at the nucleotide level with unique primers and so *emrl1*/*emrl2* expression is shown (A).

**Figure 6:**
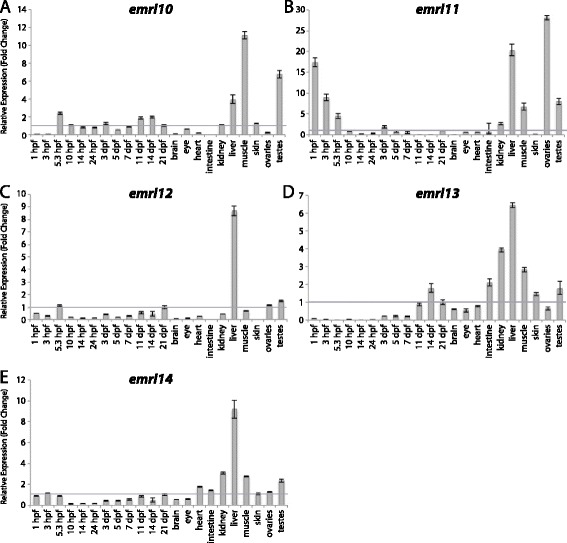
**Group II aGPCR expression data – part 3.** Relative expression of zebrafish Group II aGPCRs obtained with either high-throughput quantitative real-time PCR or conventional qPCR in a collection of 12 developmental time-points and 10 adult tissues. **(A)** *emrl10*, **(B)** *emrl11*, **(C)** *emrl12*, **(D)** *emrl13*, and **(E)** *emrl14*. Error bars display the upper (RQmax) and lower (RQmin) limits of possible relative quantification values. Fold changes shown are relative to expression at 21 dpf (denoted by gray line at y = 1).

**Figure 7:**
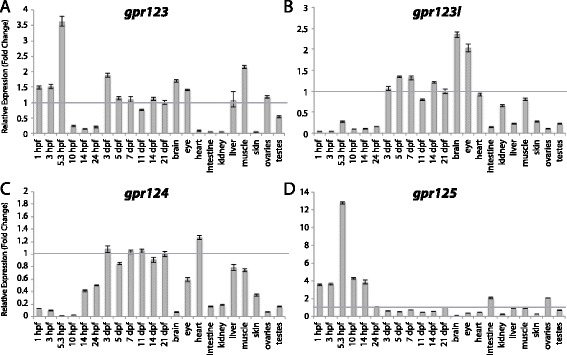
**Group III aGPCR expression data.** Relative expression of zebrafish Group III aGPCRs obtained with high-throughput quantitative real-time PCR in a collection of 12 developmental time-points and 10 adult tissues. **(A)** *gpr123*, **(B)** *gpr123l*, **(C)** *gpr124*, **(D)** *gpr125*. Error bars display the upper (RQmax) and lower (RQmin) limits of possible relative quantification values. Fold changes shown are relative to expression at 21 dpf (denoted by gray line at y = 1).

**Figure 8:**
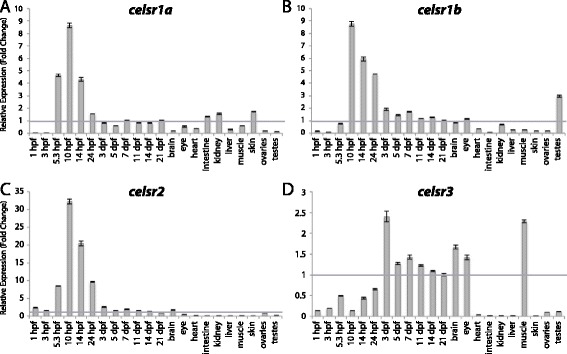
**Group IV aGPCR expression data.** Relative expression of zebrafish Group IV aGPCRs obtained with high-throughput quantitative real-time PCR in a collection of 12 developmental time-points and 10 adult tissues. **(A)** *celsr1a*, **(B)** *celsr1b*, **(C)** *celsr2*, **(D)** *celsr3*. Error bars display the upper (RQmax) and lower (RQmin) limits of possible relative quantification values. Fold changes shown are relative to expression at 21 dpf (denoted by gray line at y = 1).

**Figure 9:**
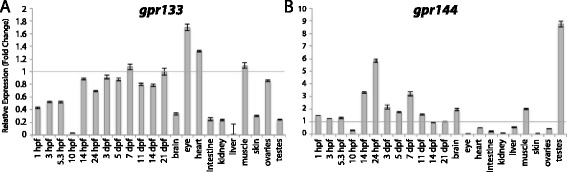
**Group V aGPCR expression data.** Relative expression of zebrafish Group V aGPCRs obtained with high-throughput quantitative real-time PCR in a collection of 12 developmental time-points and 10 adult tissues. **(A)** *gpr133*, **(B)** *gpr144*. Error bars display the upper (RQmax) and lower (RQmin) limits of possible relative quantification values. Fold changes shown are relative to expression at 21 dpf (denoted by gray line at y = 1).

**Figure 10:**
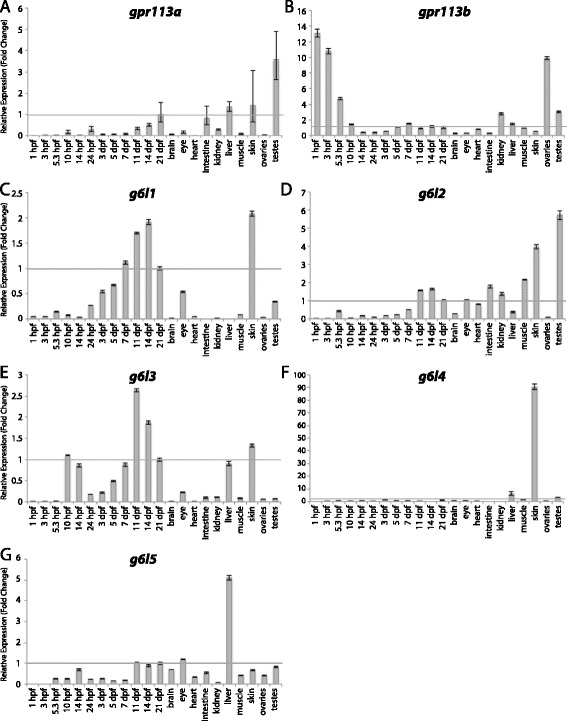
**Group VI aGPCR expression data.** Relative expression of zebrafish Group VI aGPCRs obtained with either high-throughput quantitative real-time PCR or conventional qPCR in a collection of 12 developmental time-points and 10 adult tissues. **(A)** *gpr113a*, **(B)** *gpr113b*, **(C)** *g6l1*, **(D)** *g6l2*, **(E)** *g6l3*, **(F)** *g6l4*, **(G)** *g6l5*. Error bars display the upper (RQmax) and lower (RQmin) limits of possible relative quantification values. Fold changes shown are relative to expression at 21 dpf (denoted by gray line at y = 1).

**Figure 11:**
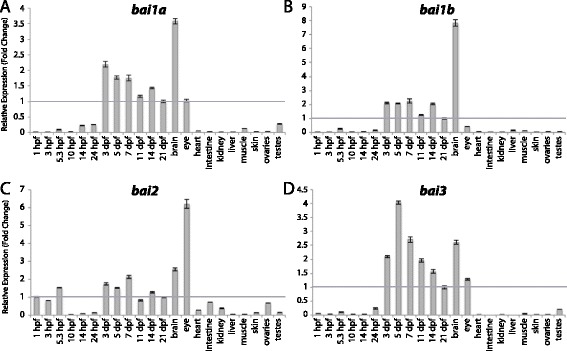
**Group VII aGPCR expression data.** Relative expression of zebrafish Group VII aGPCRs obtained with high-throughput quantitative real-time PCR in a collection of 12 developmental time-points and 10 adult tissues. **(A)** *bai1a*, **(B)** *bai1b*, **(C)** *bai2*, **(D)** *bai3*. Error bars display the upper (RQmax) and lower (RQmin) limits of possible relative quantification values. Fold changes shown are relative to expression at 21 dpf (denoted by gray line at y = 1).

**Figure 12:**
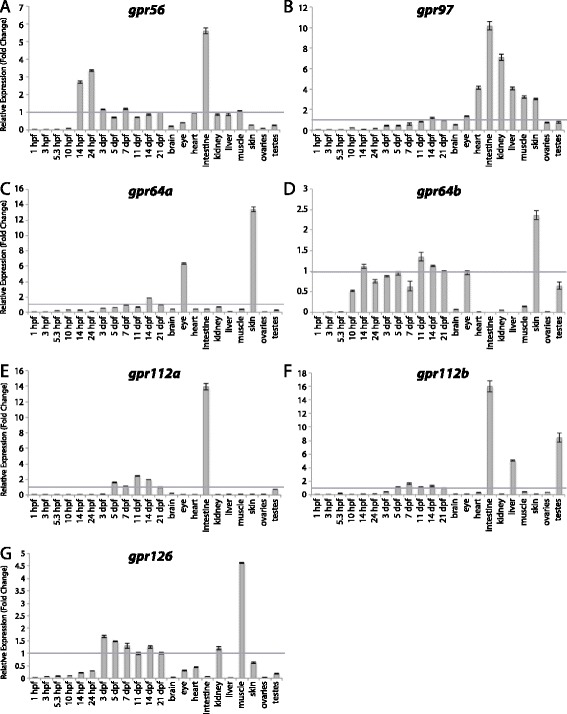
**Group VIII aGPCR expression data – part 1.** Relative expression of zebrafish Group VIII aGPCRs obtained with high-throughput quantitative real-time PCR in a collection of 12 developmental time-points and 10 adult tissues. **(A)** *gpr56*, **(B)** *gpr97*, **(C)** *gpr64a*, **(D)** *gpr64b*, **(E)** *gpr112a*, **(F)** *gpr112b*, **(G)** *gpr126*. Error bars display the upper (RQmax) and lower (RQmin) limits of possible relative quantification values. Fold changes shown are relative to expression at 21 dpf (denoted by gray line at y = 1).

**Figure 13:**
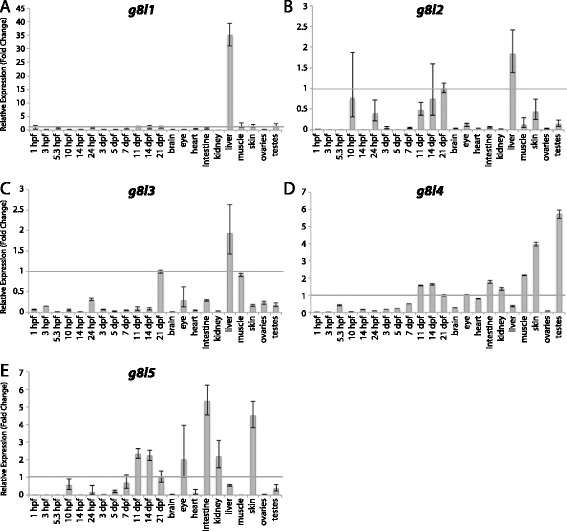
**Group VIII aGPCR expression data – part 2.** Relative expression of zebrafish Group VIII aGPCRs obtained with conventional quantitative real-time PCR in a collection of 12 developmental time-points and 10 adult tissues. **(A)** *g8l1*, **(B)** *g8l2*, **(C)** *g8l3*, **(D)** *g8l4*, **(E)** *g8l5*. Error bars display the upper (RQmax) and lower (RQmin) limits of possible relative quantification values. Fold changes shown are relative to expression at 21 dpf (denoted by gray line at y = 1).

**Figure 14:**
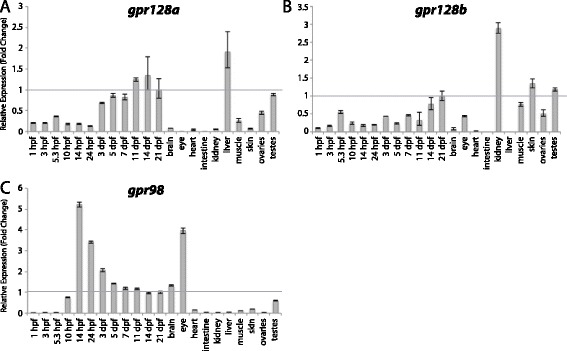
**Group IX aGPCR expression data.** Relative expression of zebrafish Group IX aGPCRs obtained with high-throughput quantitative real-time PCR in a collection of 12 developmental time-points and 10 adult tissues. **(A)** *gpr128a*, **(B)** *gpr128*b, **(C)** *gpr98*. Error bars display the upper (RQmax) and lower (RQmin) limits of possible relative quantification values. Fold changes shown are relative to expression at 21 dpf (denoted by gray line at y = 1).

Figures 3, 4, 5, 6, 7, 8, 9, 10, 11, 12, 13, and 14 show fold change in expression for each of the 9 aGPCR groups. Tissues and/or time-points for which fold changes ≤ 1 are described as “lowly expressed” or “not enriched”, fold changes between 1 – 3 are described as “slight enrichment”, and fold changes > 3-fold are described as “highly enriched”. It is important to emphasize that this method of analysis is meant to show enrichment over expression levels in the whole fish at 21 dpf, and does not depict raw expression. Therefore, an aGPCR might be expressed in and play an important role in certain cell types within a tissue even if that gene does not appear to be enriched in that tissue given these analyses. Additionally, it is possible that some of the tissue-specific expression could be due to factors such as: residual blood, tissue-resident immune cells, or fat cells. For example, the thymus, kidney, and spleen are known to be the major lymphoid organs in adult teleosts [41], so kidney analysis may represent expression in kidney tissue as well as in immune cells.

Importantly, nearly all of the zebrafish aGPCRs display expression profiles similar to their rodent and human orthologs in adult tissues [32,38], and our developmental data corroborates previous studies on individual family members in zebrafish [18-23,42-45]. We validated expression of all genes by performing RT-PCR on cDNA samples obtained from a subset of developmental time-points and adult samples (data not shown). We further validated qPCR results by comparisons to previously described whole-mount *in situ* hybridization (WISH) data for a subset of aGPCRs [42-44,46].

In zebrafish, early development proceeds in the absence of *de novo* zygotic transcription and relies upon maternal mRNA contribution. Zygotic transcription of some genes begins at approximately 2.25 hpf (128-cell stage), while for most other zygotic genes it begins after 3 hpf (1000-cell stage), as the maternal transcripts gradually become diluted [45]. Previous reports on the zebrafish maternal transcriptome suggest that 34% of all protein-coding genes are expressed exclusively as maternal transcripts, 61% are expressed both maternally and zygotically, and 5% only undergo zygotic transcription [45]. In agreement with these data, 96% of aGPCRs have at least some level of maternal expression, while only 4% of aGPCRs appear to be exclusively zygotically expressed. Interestingly, however, we did not observe any aGPCRs that were only expressed maternally (at 1 hpf), suggesting that this class of receptors is important throughout additional stages of development.

#### Zebrafish aGPCRs – group I

Group I aGPCRs are composed of the Latrophilins and Eltd1 (Figures 1, 2, and 3). The two paralogs of *lphn1*, *lphn1a* and *lphn1b*, have nearly identical expression profiles with low expression during development until 3 dpf and specific enrichment in the adult brain and eye (Figure 3A-B). In contrast to the other Group I members, *lphn2a* and *lphn2b* are enriched earlier in development during gastrulation, with no significant enrichment in adult tissues (Figure 3C-D). Interestingly, the expression profile of *lphn3* is remarkably similar to that of *lphn1a* and *lphn1b*, with the exception of slightly higher maternal and adult eye expression (Figure 3E). *eltd1* is expressed ubiquitously during development with slight enrichment maternally (1 hpf) and during gastrulation (5.3 hpf), but is highly enriched in the adult heart, liver, and muscle (Figure 3F).

#### Zebrafish aGPCRs – group II

Group II aGPCRs are composed of Cd97 and the Emrs (Figures 1, 2, and 4, 5, 6). With the exception of *cd97b*, which is slightly enriched during gastrulation and segmentation stages (Figure 4B), the *cd97* paralogs do not show enrichment during development. In contrast, all four *cd97* paralogs are highly enriched in adult muscle, and to varying degrees in the intestine and liver (Figure 4A-D). All *cd97* paralogs except *cd97d* also show some enrichment in the kidney (Figure 4A-C). Finally, *cd97a* and *cd97b* are enriched in the adult heart (Figure 4A-B), and *cd97a* and *cd97c* are enriched in testes (Figure 4A, C).

Whereas humans possess EMR1-4 and mice have homologs of EMR1 and EMR4, zebrafish possess at least 14 EMR-like proteins (Figures 1, 2, 5, and 6). Although GenBank defines some of these predicted zebrafish Emr-like genes into “*emr1-like*” or “*emr3-like*”, we did not find sufficient evidence to suggest that the zebrafish Emr-like sequences were more similar to any of the four human EMRs. However, these sequences did cluster with Group II, but distinctly from the Cd97 clade (Figure 1). Therefore, we named these genes *emr-like* followed by a number (*i.e.*, *emrl1-emrl14*).

Interestingly, although the zebrafish EMR-like proteins phylogenetically cluster together in a zebrafish-specific expansion of Group II (Figure 1), and not clearly with the human and mouse orthologs of EMR1-4, the zebrafish gene expression profiles in adult tissues resemble those of human *EMR1-3* [38] and mouse *Emr1* and *Emr4* [32]. It is important to note that two of the zebrafish Emr-like genes, *emrl1* and *emrl2*, are so similar at the nucleotide level that they could not be distinguished by unique primers that were compatible with qPCR assays. Therefore, primers were designed that could amplify *emrl1* and *emrl2* distinctly from the other 12 zebrafish *emr-like* genes, and the expression of *emrl1/emrl2* is shown on the same graph (Figure 5A). Interestingly, all but four of the zebrafish *emr-like* genes (*emrl10*, *emrl11*, *emrl12*, and *emrl14*) are lowly expressed during early development, and only begin to show very slight enrichment between 11 dpf and 21 dpf. Additionally, at least 9 of the 14 *emr-like* genes show enrichment in the adult liver (Figure 5A-B, D, F, and Figure 6A-E) and in the testes (Figures 5A-D, F, and 6A-E). In terms of specific enrichment, *emrl1* and/or *emrl2* are enriched in the brain (Figure 5A), *emrl3*, *emrl7*, and *emrl10* are also highly enriched in skeletal muscle (Figures 5B, F and 6A), and *emrl4* is slightly enriched in the skin (Figure 5C). *emrl6* and *emrl8* are very lowly expressed, showing no enrichment over 21 dpf at any stage of development or in any adult tissue (Figure 5E, G). Interestingly, *emrl11* is the only *emr-like* gene that is highly enriched during maternal transcription and in adult ovaries, in addition to its enrichment in the liver, muscle, and testes (Figure 6B). Finally, *emrl13* and *emrl14* have broader expression profiles than their fellow *emr-like* genes, with varying degrees of enrichment in the adult heart, intestine, kidney, liver, muscle, skin, and testes (Figure 6D-E).

#### Zebrafish aGPCRs – group III

Group III aGPCRs are composed of Gpr123, Gpr123L, Gpr124, and Gpr125 (Figures 1, 2, and 7). Interestingly, *gpr123* and *gpr123l* are differentially expressed both during development as well as in adult tissues. Both genes are slightly enriched at all developmental stages after 3 dpf, except at 11 dpf, as well as in the adult brain and eye (Figure 7A-B). However, *gpr123* is also enriched during maternal and early gastrulation stages (1 hpf – 5.3 hpf) and in the adult liver, muscle, and ovaries (Figure 7A). *gpr124* is ubiquitously expressed at very low levels during early development with slightly higher expression after 3 dpf and a slight enrichment in the adult heart (Figure 7C). Developmental expression data for *gpr125* are consistent with previous reports in zebrafish, which show that it is highly enriched during early development [22] and then ubiquitously expressed at very low levels at later larval stages and in adult tissues (Figure 7D).

#### Zebrafish aGPCRs – group IV

Group IV aGPCRs are composed of the Celsr proteins (Figures 1, 2, and 8). Consistent with previous studies in zebrafish [20,21,23], we found that all Group IV aGPCRs except *celsr3* are highly expressed during gastrulation and segmentation stages and then expressed at lower levels during later development and in adult tissues (Figure 8A-C). In contrast, *celsr3* is lowly expressed until 3 dpf, and then highly enriched in the brain, eye, and skeletal muscle in adults (Figure 8D).

#### Zebrafish aGPCRs – group V

Group V aGPCRs are composed of Gpr133 and Gpr144 (Figures 1, 2, and 9). *gpr133* is expressed ubiquitously at low levels both during development and in adult tissues, with the exception of slight enrichment in the adult eye and heart (Figure 9A). In contrast, *gpr144* is slightly maternally enriched and highly enriched beginning at segmentation stages (14 hpf). *gpr144* is also slightly enriched in the adult brain and skeletal muscle and highly enriched in adult testes (Figure 9B).

#### Zebrafish aGPCRs – group VI

Group VI aGPCRs are composed of Gpr113a, Gpr113b, and five Group VI-like genes (G6L1-5) that appear to be zebrafish specific (Figures 1, 2, and 10). We note that the sequences for *g6l1-5* are labeled as “predicted *gpr110*-like” or “predicted *gpr116*-like” in GenBank; however, there was not sufficient evidence based on sequence similarities, protein structure, or phylogenetics to confidently call these genes homologs of mammalian Gpr110 and Gpr116. With the exception of *gpr113b*, and a very slight enrichment of *g6l3* at 10 hpf, Group VI aGPCRs are expressed at low levels during early development. *gpr113a* is lowly expressed at all stages of development, but is enriched in the adult liver, skin, and testes (Figure 10A). *gpr113b* is highly expressed maternally and during early gastrulation; additionally, *gpr113b* is enriched in the adult kidney, liver, ovaries, and testes (Figure 10B). *g6l1* and *g6l3* are slightly enriched after 7 dpf and in the adult skin (Figure 10C, E). *g6l2* is slightly enriched after 11 dpf and in the adult intestine, kidney, and muscle, and is highly enriched in skin and testes (Figure 10D). *g6l4* is expressed very lowly during development; however, it is highly and specifically enriched in the adult skin (Figure 10F). *g6l5* is not significantly enriched at any stage of development, but is highly enriched in the adult liver (Figure 10G).

#### Zebrafish aGPCRs – group VII

Group VII aGPCRs are composed of the BAIs (Figures 1, 2, and 11). Expression analysis of Group VII aGPCRs is consistent with previous reports in zebrafish [42] and mammals [32,38,47,48], with significant enrichment in the brain for all members (Figure 11A-D). Additionally, with the exception of *bai2*, all of the Group VII aGPCRs do not show enrichment during development until 3 dpf. Interestingly, *bai2* also shows slightly enriched maternal expression, and is the only group member to show higher enrichment in the adult eye than in the adult brain (Figure 11C).

#### Zebrafish aGPCRs – group VIII

Group VIII aGPCRs are composed of Gpr56, Gpr97, Gpr64a, Gpr64b, Gpr112a, Gpr112b, and Gpr126, as well as five zebrafish-specific Group VIII-like genes (G8L1-5) (Figures 1, 2, 12, and 13). With the exception of *gpr56*, *gpr64b*, and *g8l2*, Group VIII aGPCRs are lowly expressed during early development, and do not show significant enrichment until 3–5 dpf. In contrast, *gpr56* is highly enriched at segmentation stages (Figure 12A). *gpr56*, *gpr97*, *gpr112a*, *gpr112b*, and *g8l5* are all enriched in the adult intestine (Figures 12A-B, E-F and 13E), consistent with observations in the rat gastrointestinal tract for *Gpr56*, *Gpr97*, and *Gpr112* [49]. Additionally, *gpr97* is enriched in the adult heart, kidney, liver, muscle, and skin (Figure 12B). Both paralogs of *gpr64* are enriched in the skin (Figure 12C-D), but interestingly, *gpr64a* is also highly enriched in the eye (Figure 12C). Similarly, while both paralogs of *gpr112* are highly and specifically enriched in the adult intestine (Figure 12E, F), *gpr112b* is also enriched in the liver and testes (Figure 12F). In adult tissues, *gpr126* is slightly enriched in the kidney and highly enriched in skeletal muscle (Figure 12G).

The genes that make up the zebrafish-specific expansion of Group VIII show broadly similar expression profiles to other Group VIII members, with low expression during development and specific enrichment in a few adult tissues (Figure 13A-E). *g8l1*, *g8l2*, and *g8l3* are all specifically enriched in the adult liver (Figure 13A-C). *g8l4* and *g8l5* are slightly enriched after 11 dpf, as well as in the adult intestine, kidney, and skin (Figure 13D-E). Additionally, *g8l4* is slightly enriched in skeletal muscle and highly enriched in testes (Figure 13D), while *g8l5* is also enriched in the adult eye (Figure 13E).

#### Zebrafish aGPCRs – group IX

The final group of aGPCRs consists of Gpr98, Gpr128a, and Gpr128b (Figures 1, 2, and 14). However, it should be noted that these genes do not phylogenetically cluster together into a clade, but considered together here because they do not cluster distinctly with any of the other eight groups (Figure 1). Interestingly, *gpr128a* and *gpr128b* are both expressed very lowly during early development, but are differentially enriched specifically in the adult liver and kidney, respectively (Figure 14A-B). *gpr98* is highly enriched during late gastrulation stages as well as in the adult eye (Figure 14C). These results are consistent with a role of *GPR98* in retinal disease and Usher syndrome in humans [46].

## Conclusions

**Figure 15:**
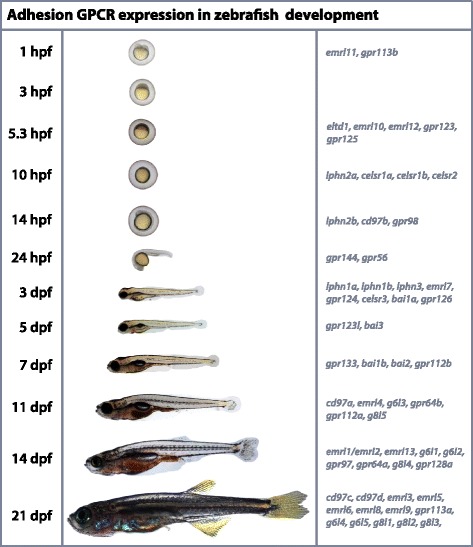
**A summary of aGPCR peak enrichments during zebrafish development.** Light microscope images of wild-type AB* zebrafish embryos/larvae/fry are shown next to the corresponding developmental stages that were used in the expression studies. All images are shown at the same magnification to show relative size as the animal ages. Zebrafish aGPCR gene names are listed next to the developmental stage at which their expression was most highly enriched over 21 dpf (right column).

Here we have shown that there are at least 59 aGPCRs in the zebrafish genome that represent homologs of 24 of the 33 aGPCRs found in humans. Phylogenetic analysis of the 7TM suggests that the zebrafish aGPCRs cluster closely with their mammalian homologs and separate into the same nine groups as previously described for the human aGPCR repertoire [4]. In adult tissues, zebrafish aGPCR expression profiles are quite similar to those previously described for aGPCRs in rodents [32] and humans [38]. This study provides the first quantitative description of the expression profiles of this gene family over an extensive developmental time-course. Importantly, our data also agrees with previously reported WISH data for a subset of aGPCRs at a few different developmental stages [18-23,42-45]. A summary of aGPCR peak enrichments during zebrafish development is shown in Figure 15. Interestingly, none of the zebrafish aGPCRs were most highly enriched at 3 hpf (1000-cell stage), when zygotic transcription is beginning; however, whether or not there is any functional significance to this observation is unknown.

Our data suggests it is likely that aGPCRs play important roles in fish as at least 59 members are found in a single teleost species, compared with 33 members in humans and 31 in mouse and rat. The majority of these proteins are classified as “orphan” receptors meaning the ligand(s) is unknown, and for most family members, precise biological functions remain mysterious. Despite our incomplete understanding of aGPCRs, they have been implicated in many crucial physiological processes, both during early development as well as in adult tissues, including but not limited to the role(s) of Celsr proteins [20,21] and Gpr125 [22] in gastrulation, Celsr1-3 in the migration of facial branchiomotor neurons during hindbrain development [50], Gpr126 in the development of the ear [19] and Schwann cells [18,51-53], Gpr124 in regulating CNS angiogenesis [54], CD97 in leukocyte trafficking and adaptive T-cells responses [55], EMR1 in the production of CD8+ cells [56], GPR64 in male infertility [57], and BAI1-3 in inhibiting angiogenesis in the brain [47,48].

The importance of aGPCRs in human health is further underscored in diseases in which they are disrupted, such as bilateral frontoparietal polymicrogyria (*GPR56*) [58,59], Usher Syndrome (*GPR98*) [60-62], glioblastomas (*BAI1*) [63,64], susceptibility to brain arteriovenous malformation (*GPR124*) [65], and breast cancer metastasis (*GPR116*) [66].

Our data likely represent the majority of zebrafish aGPCRs, although it is difficult to be sure that we have identified every aGPCR in the zebrafish repertoire for several reasons. First, zebrafish possess 26,206 protein-coding genes, more than any previously sequenced vertebrate species [67], and yet the zebrafish genome is not as well sequenced or as well annotated as the human and mouse genomes. The release of Zv9 significantly improved the quality of the zebrafish reference sequence and demonstrated that the zebrafish genome has an overall repeat content of 52.2%, the highest reported in any vertebrate species to date [37]. However, the highly repetitive nature of the zebrafish genome makes correct annotation of different gene duplicates without manual curation challenging. Additionally, we only included full-length aGPCR sequences that have the appropriate hallmark aGPCR domains (GPS and 7TM) in this study. Notably, we also identified several partial putative aGPCR sequences that appear to only contain some of the domains found in the N-termini of these proteins (ENSDARG00000075133, ENSDARG00000075899, ENSDARG00000074366, ENSDARG00000088231, XM_003197748.2, XM_005163532.1, XM_005163531.1, XM_005171021.1, XM_005163529.1). Further analysis will be necessary to determine if these sequences are pseudogenes, actually belong to one of the full-length sequences (perhaps as misannotated splice isoforms), or if their remaining domains have not yet been found and annotated. It is also important to note that our approach of using whole animals for developmental time-points and whole adult organs has limited sensitivity and might not reveal instances in which an aGPCR functions, or is expressed, in very specific and/or scarce cell types. For example, *Gpr126* is expressed in Bergmann glia of the cerebellum in mouse [68], but in zebrafish, *gpr126* is not enriched in whole brain. Additionally, several aGPCRs are known to be highly expressed and to play critical roles in immune cells; however, the relative abundance of resident immune cells in different tissues is unknown in zebrafish. Therefore, we cannot rule out the possibility that some of the expression signal of any tissue might be in part due to resident immune cells or immune cells from residual blood in or on the tissue at the time of sample processing. Future work, including cell type-specific expression analyses, global and cell-specific mutant studies, and additional curation of the zebrafish reference genome can address these limitations.

In sum, aGPCR biology is a highly active field of research that is also attractive for its potential implications in human health. Approximately 30% of newly introduced drugs target GPCRs [69], suggesting that aGPCRs may represent potentially novel therapeutic targets for a wide variety of human pathologies. Zebrafish are genetically tractable vertebrates that can rapidly produce large numbers of progeny that are transparent during early development. This allows for straightforward genetic manipulation and the use of live animal imaging techniques for phenotypic analysis. The recent advent of TALEN [70] and CRISPR-Cas [71] technologies has also made genome editing very fast and efficient in zebrafish. Additionally, zebrafish are highly amenable to drug screens, and have already proven to be a useful model for the study of aGPCR biology. Therefore, the provision of the zebrafish aGPCR repertoire and their expression profiles herein should allow for significant advancement of aGPCR research in the zebrafish community which will eventually result not only in our further understanding of this unique GPCR family, but may also lay the foundation for future work to modulate these proteins in human disease.

## Methods

### Alignments and phylogenetic analysis

Multiple sequence alignments analyzed in this study were generated using MAFFT version 6 (http://mafft.cbrc.jp/alignment/server/) with BLOSUM62 as the scoring matrix and using option E-INS-I. [72]. The evolutionary history of the 7TM domain of zebrafish, mouse, and human aGPCRs was inferred using the Maximum Likelihood method based on the Jones-Taylor-Thornton (JTT) matrix-based model using MEGA5 [73]. Amino acid sequences of the 7TM domains were obtained from GenBank or by using protein domain prediction software [74,75] for those zebrafish aGPCR sequences not found in GenBank. The bootstrap consensus tree inferred from 1000 replicates [76] is shown in Figure 1. To account for input-order bias, similar trees were made with 5 different randomized alignments. Importantly, changing the input order did not dramatically alter tree structure under the following analysis parameters. Branches corresponding to partitions reproduced in less than 50% bootstrap replicates are collapsed. Initial tree (s) for the heuristic search were obtained by applying the Neighbor-Joining method to a matrix of pairwise distances estimated using a JTT model [73]. A discrete Gamma distribution was used to model evolutionary rate differences among sites (5 categories (+G, parameter = 2.2124)). The rate variation model allowed for some sites to be evolutionarily invariable ([+I], 0.6155% sites). The analysis involved 123 amino acid sequences. All ambiguous positions were removed for each sequence pair. There were a total of 339 positions in the final dataset, and all sites were taken into account for phylogenetic tree construction.

The topology inferred from ML analysis was verified using the using the Bayesian approach implemented in MrBayes version 3.2. Markov Chain Monte Carlo (MCMC) analysis was used to estimate the posterior probabilities (PP) and branch lengths of the trees. The best amino acid substitution model was determined using a mixed model as implemented in MrBayes and the gamma shaped model was used to estimate the variation of evolutionary rates across sites (lset rates = gamma). The Bayesian analysis included two independent MCMC runs, where each MCMC run uses 4 parallel chains composed of three heated and one cold chain. Each Markov chain was started from a random tree and was set to run for 3,000,000 generations and every hundredth tree was sampled. The convergence of the two independent MCMC runs was tested using diagnostic frequency generations and diagnostics were calculated for every 1000 generations. A stop rule was applied (standard deviation of split frequencies, 0.01) to terminate the MCMC generations. The first 25% of the sampled trees were discarded to ensure that the parameter estimates were only made from data drawn from the distributions that were derived after the MCMCs had converged. Thereafter a consensus tree was built from the remaining 75% of the sampled trees with the MrBayes sumt command using the 50% majority rule method. The phylogenetic tree was drawn in FigTree 1.3.1 (http://tree.bio.ed.ac.uk/software/figtree/).

### Prediction of zebrafish aGPCR domain architecture

The domain architectures of zebrafish aGPCRs were predicted using the Conserved Domain Search service (CD-Search) [33], Pfam search [34], and InterProScan [35], with default settings. CD-Search employs a RPS-BLAST (Reverse PSI-BLAST) search strategy and aligns the query sequence against a database containing PSSMs (position-specific scoring matrices) of protein domain models. Similarly, the Pfam search engine pairwise aligns the query sequence against a Pfam-A database of manually curated profile HMMs (Hidden Markov Model) built using the HMMER3 software package. The Pfam database ensures better predictions and sensitivity through curated Pfam-A entries built from high quality alignments with gathering threshold [34]. This curated threshold is set for each family/domain to prevent false positives from being included in the multiple sequence alignments that are used to make the HMM profile of a protein family/domain [34]. On the other hand, InterProScan integrates the predictions from an array of databases/search engines and capitalizes on their individual strengths to provide a relatively reliable protein family/domain (s) annotation for a given sequence [35]. To ensure reliable predictions of potential domains and repeats of zebrafish aGPCRs, all 59 aGPCRs were searched using these three search engines employing varied strategies and database resources. The predicted domain architectures are illustrated in Figure 2.

### Zebrafish husbandry and sample procurement

All experimental procedures involving zebrafish were performed in compliance with Washington University’s institutional animal protocols. All samples were from wild-type (AB*) zebrafish. Embryos were raised and staged using standard methods [77]. Representative individuals at each developmental stage are pictured in Figure 15. Embryos 24 hpf or younger were imaged in egg water while larvae/fry over 3 dpf were anaesthetized in 0.24% tricaine in egg water to minimize animal movements during imaging. All individuals were placed in a small amount of liquid in a 9-well clear glass spot plate and light microscope images were obtained using a Zeiss SteREO Discovery V8. For expression studies, the different developmental stages were collected from pools of embryos from multiple pairwise matings on two separate occasions. The parents were then used for the collection of adult tissues at 6 months of age. Prior to collection, zebrafish embryos/larvae were anesthetized in 0.24% tricaine. Prior to dissection, adult zebrafish were humanely euthanized by submersion in ice-cold water. Dissections were performed as previously described [78], and all tissues were rinsed briefly in 1X PBS prior to freezing in an attempt to minimize blood and fat cell contamination. For all developmental stages, two separate pooled samples were collected to control for slight daily variations in egg quality. RNA was extracted separately from each pool (see section on *RNA extraction*), and all of the RNA for a given time point was combined prior to cDNA synthesis (see section on *Reverse Transcription*). At all stages up through 7 dpf, 25 dechorionated embryos/larvae were pooled in each of the two samples, for a total of 50 embryos/larvae. For the 11 dpf time point, the two pools consisted of 15 larvae and 20 larvae for a total of 35 larvae. Finally, 10 larvae/fry were pooled in each of the two replicates for the 14 dpf and 21 dpf time points, for a total of 20 larvae/fry represented in the final pooled RNA samples. All adult tissues were collected and pooled from 4 animals (2 males and 2 females) with the exception of reproductive organs, which were collected and pooled from 3 animals of the appropriate sex. After collection, all samples were rinsed briefly in 1X PBS, immediately flash-frozen in liquid nitrogen, and stored at −80°C until RNA extraction was performed. For the developmental samples, egg-water was removed just prior to submersion in liquid nitrogen.

### RNA extraction

Total RNA was extracted from each of the two separate pools for all developmental stages, as well as the adult samples using standard methods [79]. Briefly, TRIzol (Life Technologies, Carlsbad, CA) was added to the frozen tissue samples, which were then homogenized using a combination of vortexing, disruption with a plastic-tipped electric homogenizer, and passage through syringe and successively smaller needles (22.5 g and 27 g) ten times each.

### Reverse transcription

Prior to reverse transcription, the two separate RNA samples for each developmental time point were combined. Total RNA (1.0–5.0 μg) was then reverse transcribed in 20 μl using the High Capacity cDNA Reverse Transcription Kit (Applied Biosystems, Carlsbad, CA) using random hexamers. The reaction mixture was incubated for 10 min at 25°C, 120 min at 37°C, and for 5 min at 85°C, as per the instructions from the manufacturer. Reverse transcription reactions were diluted 5–10-fold prior to qPCR. To control for genomic DNA contamination, a no reverse transcriptase reaction (RT-) was also performed for each RNA sample.

### Assay design and quality control

If possible, qPCR assays were designed to amplify 100-150 bp of the 7TM domain and oligonucleotide primers were designed to span exon-intron boundaries, as assessed by alignment of cDNA sequences to genomic regions (alignments generated using Sequencher software). All primers were 20–29 nucleotides in length, with approximate melting temperatures of 62°C, and were manufactured by Integrated DNA Technologies (IDT, Coralville, IA). BLAST searches were performed with every primer to ensure specificity of binding. All primers BLASTed specifically to the appropriate gene and the corresponding chromosomal region (Query Coverage = 100%, Identity = 100%) with no other “hits” having a query coverage or identity greater than 85%. All gene accession numbers, their corresponding primer sequences, standard curve slopes, and R^2^ values are provided in Additional file 1.

Standard curves and qPCR assays for the 9 new aGPCRs found after the release of Zv9 (*gpr113a*, *emrl3*, *emrl5*, *emrl8*, *emrl9*, *g8l1*, *g8l2*, *g8l3*, *g8l5*) were performed using conventional qPCR in 10 μl volumes in 384-well plates. Standard curve assays were run in duplicate using a 4-point serial dilution beginning with 200 ng 21 dpf cDNA. These assays were performed in triplicate on a ViiA7 (Applied Biosystems) qPCR machine with 2X SsoFast Evagreen Supermix (Applied Biosystems), and an assay concentration of 100 nM. To control for any genomic DNA (gDNA) contamination in the cDNA samples, RT- controls were performed for all primer sets on all samples. Cycling parameters were 95°C (10 min) followed by 40 cycles of 95°C (15 s), then 60°C (1 min). Melting curve analysis was completed as follows: 95°C (15 s), 60°C (1 min), and a progressive increase up to 95°C (0.5°C/min). Calculations of slopes and R^2^ values were performed with the ViiA7 software.

### Quantitative real-time PCR

High-throughput qPCR was performed at the Washington University in St. Louis Genome Technology Access Center (GTAC) using the 96X96 Dynamic Array Interfluidic circuit for the microfluidic BioMark^TM^ system (Fluidigm Corporation, San Francisco, CA) [39]. All assays were run in all cDNAs in triplicate, in addition to an RT- control for each sample. Specific-target amplification, the sample mix, assay mix, and qPCR conditions were performed as previously described [39] with the following modifications: pre-amplification was performed on 37.5 ng cDNA from zebrafish samples.

qPCR data was analyzed using Microsoft Excel. Relative expression was calculated using the ΔΔCt method [40]. All Ct values derived from reverse-transcribed (RT+) samples were then corrected to remove signal from gDNA (RT- controls) using the following formula: Ct_RNA_ = −log2 (2^-CtRT+^ - 2^-CtRT-^). All tissues were normalized to a stably expressed control gene, *importin8* (*ipo8*), to control for variation in amount of starting material (ΔCt). *importin8* was chosen from among 3 housekeeping genes tested (*ipo8*, *tbp*, and *gapdh*) because it only has one known transcript and it is expressed at stable levels in all developmental stages and tissues. ΔΔCt was then calculated relative to expression at 21 days post fertilization (dpf). Relative expression (RQ), or fold change (2^-ΔΔCt^), is shown in Figures 3, 4, 5, 6, 7, 8, 9, 10, 11, 12, 13 and 14. Error bars depict RQmax and RQmin, which are the maximum and minimum limits of possible RQ values based on the standard error of the ΔCt values.

All qPCR experiments and provision of data in this study were conducted in line with the guidelines for the minimum information for publication of quantitative real-time PCR experiments (MIQE) [80].

## Availability of supporting data

All supporting data are available as additional files.

## Additional files

Additional file 1:
**Excel spreadsheet containing database accession numbers, primer sequences, primer slopes, and primer R** ^**2**^ **values for all zebrafish aGPCRs and reference genes used in the qPCR studies.**
Additional file 2:
**Phylogenetic tree topologies obtained using Bayesian analysis for Group I (left) and Group VIII (right) independently.** These trees were used to confirm the nomenclature for zfLphn1b and zfGpr56 as the zebrafish homologs of human LPHN1 and human GPR56, respectively. Additional file 3:
**Excel spreadsheet containing raw qPCR data for all full-length zebrafish aGPCRs.**

## Abbreviations

7TM: Seven-transmembrane domain
aGPCR: Adhesion G protein-coupled receptor
CTF: C-terminal fragment
CRISPR: Clustered regularly interspaced short palindromic repeat
dpf: Days post-fertilization
GAIN: GPCR autoproteolysis-inducing domain
GPCR: G protein-coupled receptor
GPS: GPCR proteolytic site
GRAFS: Glutamate, rhodopsin, adhesion, frizzled/taste2, and secretin
hpf: Hours post-fertilization
NTF: N-terminal fragment
qPCR: Quantitative real-time PCR
TALEN: Transcription activator-like nuclease
