# Supplementary material for: Defining the gene repertoire and spatiotemporal expression profiles of adhesion G protein-coupled receptors in zebrafish
Source: BMC Genomics. 2015 Feb 8;16(1):62. doi: 10.1186/s12864-015-1296-8 (PMC4335454; doi:10.1186/s12864-015-1296-8)

Additional File 2: Bayesian topology supporting homologous relationships of zfGpr56 and zfLphn1b

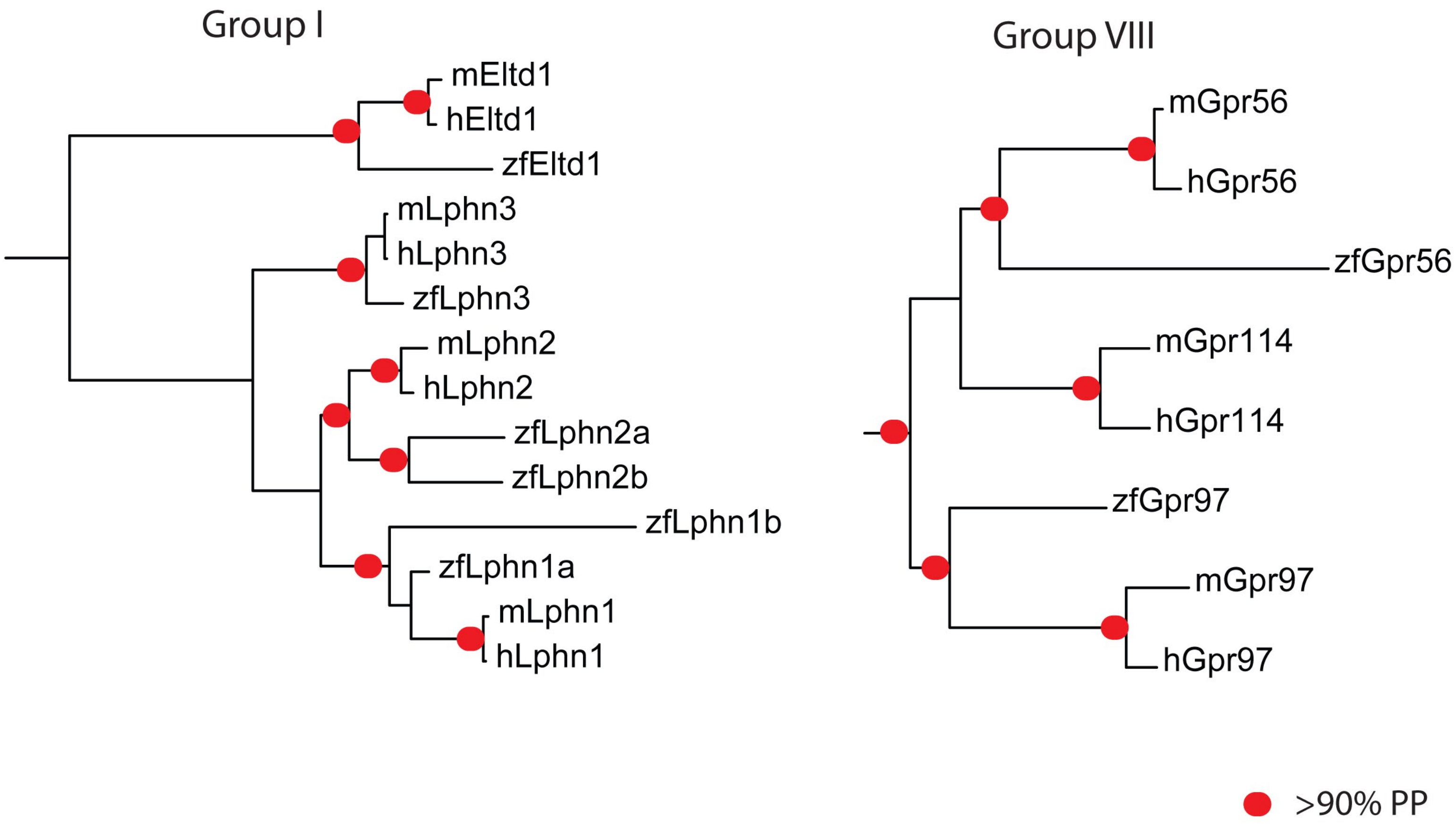

Supplement: Additional file 2: — Phylogenetic tree topologies obtained using Bayesian analysis for Group I (left) and Group VIII (right) independently. These trees were used to confirm the nomenclature for zfLphn1b and zfGpr56 as the zebrafish homologs of human LPHN1 and human GPR56, respectively. [file 12864_2015_1296_MOESM2_ESM.pdf]
